# Participation of Lattice Oxygen in Perovskite Oxide as a Highly Sensitive Sensor for p-Phenylenediamine Detection

**DOI:** 10.3390/molecules28031122

**Published:** 2023-01-22

**Authors:** Juan He, Xiaomin Xu, Hainan Sun, Tengfei Miao, Meisheng Li, Shouyong Zhou, Wei Zhou

**Affiliations:** 1 Jiangsu Collaborative Innovation Center of Regional Modern Agriculture & Environmental, School of Chemistry and Chemical Engineering, Huaiyin Normal University, No. 111 West Changjiang Road, Huaian 223300, China; 2 State Key Laboratory of Materials-Oriented Chemical Engineering, College of Chemical Engineering, Nanjing Tech University, Nanjing 210009, China; 3 WA School of Mines: Minerals, Energy and Chemical Engineering (WASM-MECE), Curtin University, Perth, WA 6102, Australia; 4 Department of Materials Science and Engineering, Korea Advanced Institute of Science and Technology (KAIST), Daejeon 34141, Republic of Korea

**Keywords:** lattice oxygen, phosphorus-doped SrCo_0.95_P_0.05_O_3−δ_, p-phenylenediamine (PPD), electrochemical sensors, hair dyes

## Abstract

The harmful effects on the human body from p-phenylenediamine (PPD) in hair dyes can cause allergies and even cancer. Therefore, it is particularly important to accurately control and detect the content of PPD in our daily products and environment. Here, a small amount of non-metallic elemental P doped in perovskite oxide of SrCoO_3−δ_ (SC) forms a good catalytic material, SrCo_0.95_P_0.05_O_3−δ_ (SCP), for PPD detection. The improved performance compared with that of the parent SC can be attributed to three contributing factors, including a larger amount of highly oxidative oxygen species O^22−^/O^−^, better electrical conductivity, and more active sites on the P^5+^-oxygen bonds of SCP. Moreover, the lattice oxygen mechanism (LOM) with highly active species of lattice O vacancies and adsorbed –OO for electrocatalytic oxidation of PPD by the SCP/GCE (glass carbon electrode) sensor is proposed in our work. More importantly, the SCP/GCE sensor exhibits good stability, a low limit of detection, and high reliability (error < 5.78%) towards PPD determination in real samples of hair dyes, suggesting the substantial research potential for practical applications.

## 1. Introduction

With improved living standards and diverse fashion culture, people have paid more attention to and more money on their external image than in previous decades. For example, unique tattoos and gorgeous hair colors are not only a show of personality, but also symbols of the fashion world. The use of hair dyes has become a globally popular way to change external appearance, enhance self-confidence, and catch up with the current trends. However, the safety of the related products has also attracted increasing attention. There are many types of hair dye products, among which oxidized hair dyes are one of the most popular because of their excellent coloring effects, long color retention, and many color options. In recent years, many reports have proven that the dye intermediate components of oxidative hair dyes, such as p-phenylenediamine (PPD), p-aminophenol, and their derivatives, pose great health threats to humans. The dye intermediate may cause allergic reactions including itching, papules, and even blindness if carelessly splashed into the eye [1]. As a result, many countries have clear regulations on the content of different dye intermediates in cosmetics. For example, in China, the maximum content of PPD in cosmetics should not exceed 6% [2]. Research on PPD safety and the detection of its content has aroused great interest, since PPD is one of the most widely used dye intermediates in the cosmetics and dyeing industries. Once excess amounts of PPD are discharged into the natural environment and finally enter the human body, this may cause non-Hodgkin’s lymphoma, bladder cancer, kidney damage, bronchial asthma, and other diseases [3,4]. Therefore, the determination of PPD content in hair dyes, industrial wastewater, and the living environment is vital.

Compared with traditional methods (e.g., spectrophotometry, fluorimetry method, and high-performance liquid chromatography [5,6,7]), electrochemical detection technology has broader research potential because of its high sensitivity, simple operation, and low cost [8,9,10]. In addition, the good conductivity, large active surface area, and high catalytic activity of nanostructured carbon-based materials and metal compounds make them superior electrocatalytic candidates compared to other materials [11,12,13,14,15,16,17]. For instance, the self-doped TiO_2_ nanotube electrode (P-TiO_2_NTs) has good performance in the case of Fe(CN)_6_^4−^ and PPD oxidation [12]. After a simple cathodic polarization of TiO_2_ nanotube arrays, the limit of detection (LOD) is 0.056 µM, and the sensitivity is 1456 µA mM^−1^ cm^−2^ (0.5–5µM). Moreover, HRP (horseradish peroxidase)/NPG (nanoporous gold)/GCE is an excellent biosensor for detecting PPD, mainly because the three-dimension structure of NPG has good conductivity, biocompatibility, and co-catalytic activity of NPG and HRP [18]. However, the semiconductors of *n*-type of TiO_2_NTs with poor electrical conductivity and the use of precious metals of NPG limit their practical application. Among these, perovskite oxides have received increasing attention in many fields, such as solid oxide fuel cells, water splitting, electrochemical sensors, and water treatment [19,20,21,22,23,24,25], due to their flexible structure and composition, high intrinsic catalytic activity, good conductivity and biocompatibility, and oxygen ion mobility. 

The general formula of perovskite oxides is ABO_3_, where A-site cations are alkaline earth metals or rare earth metals, and B-site cations are transition metals. The groups of Rossmeisl and Koper evaluated the performance of LaMO_3_ and SrMO_3_ (where M atoms are B-site cations) using density functional theory (DFT) and found that SrCoO_3_ has a better catalytic activity [26,27,28]. In our previous work, Sr in SrCoO_3_ was replaced with Pr to obtain Pr_1−x_Sr_x_CoO_3−δ_ (x = 0, 0.2, 0.4, 0.6, 0.8, and 1), which showed a high electrocatalytic performance for PPD detection by producing the intermediate of HO_2_^-^ during the oxygen reduction reaction (ORR) process [29]. Although PSC82 had proven to be the best catalyst for PPD due to the highest HO_2_^-^ yield, the structural characteristics and advantages of perovskite materials have not been fully demonstrated and explored. Moreover, doping with non-metal elements to promote the structural stability of perovskites has attracted extensive research attention in recent years. For instance, the doping of P, S or Si in Ba_2_In_2_O_5_ not only transforms its structure from an ordered brownmillerite-type structure to a new disordered perovskite structure, but also promotes conductivity in the new structure [30,31]. However, room temperature electrocatalytic properties of perovskite oxides containing these non-metallic elements remain to be exploited [32]. 

In this work, the perovskite-type oxide of SrCo_0.95_P_0.05_O_3_ (SCP) was obtained by doping non-metallic elemental P in SrCoO_3−δ_ (SC). SCP shows excellent performance for PPD determination, which was mainly attributed to its good electrical conductivity, large content of highly oxidative oxygen species (O_2_^2−^/O^−^), and high activity oxygen intermediates with adsorbed –OO and lattice O vacancies that can be ascribed to the lattice oxygen mechanism (LOM). Herein, LOM can also be used to explain that SCP with P^5+^ doping could produce more active sites on the strong P^5+^-oxygen bonds and, thus, result in a better electrocatalytic effect on PPD oxidation. 

In addition, the small amount of P doping is beneficial to stabilize the tetragonal superstructure of SCP and hinders the formation of a surface amorphous layer caused by the leaching of Sr, thereby significantly enhancing the stability of SCP during the detection process. Additionally, the results of PPD electrochemical sensing detection show that the SCP/GCE (glass carbon electrode, 0.196 cm^2^) sensor is superior to the SC/GCE undoped sensor. Additionally, a real sample test of hair dye also confirmed that SCP/GCE exhibits high accuracy and recovery.

## 2. Results and Discussion

### 2.1. Characterization of the SC and SCP 

**Figure 1:**
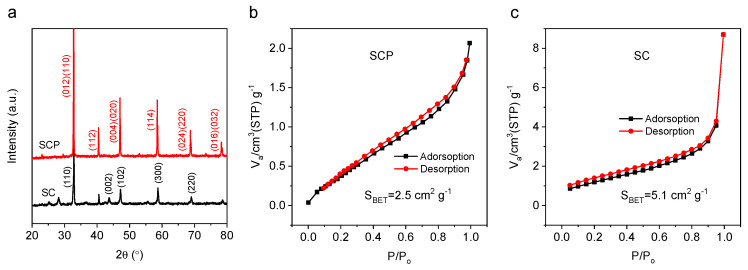
XRD (**a**) and nitrogen adsorption–desorption isotherms of SCP (**b**) and SC (**c**).

The crystal structures of the perovskite oxides were characterized by room temperature powder X-ray diffraction (XRD, Rigaku Smartlab 3KW). According to our previous works, when too much Co (i.e., over 7 mol%) was replaced by P, the impurity phase of Sr_5_(PO_4_)_3_OH could be observed [33,34]. In contrast, if the amount of P doped was less than 2 mol%, then the SC structure would not be stabilized. Therefore, a moderate amount of Co substituted by P, such as 5 mol%, can form a stabilized phase structure of SCP. According to XRD patterns (Figure 1a), the characteristic peaks of SCP and SC (at 2θ = 32.8, 40.5, 47.1, and 58.6°) show that SC presents a hexagonal phase, while SCP (5 mol% of P replacing the B-site cation of Co in SC) exhibits a pure tetragonal phase (*P4/mmm* space group), both of which are consistent with the reported work [33]. Additionally, according to the condition of the Goldschmidt tolerance factor (t) (Equation (1)) and charge neutrality criterion, the high oxidation state of P^5+^ doped in the B-site of SC can result in a part of Co^4+^ being reduced to Co^3+^ (the ionic radius of Co^3+^ is larger than Co^4+^), which increases the value of $r_{B}$ and hence makes the t value of SCP closer to 1 than that of SC (t > 1). Moreover, from the perspective of energy minimization, P^5+^ in the octahedral site of BO_6_ favors the formation of the corner-sharing octahedral configuration, which has a smaller repulsive force than face-sharing octahedra of SC. Therefore, based on the above results and discussions, the conclusion is that 5 mol% of P doping is beneficial to stabilize the tetragonal structure of SCP.

The Goldschmidt tolerance factor (*t*) [35]:

(1) $$t=\frac{r_{A}+r_{O}}{\sqrt{2}(r_{B}+r_{O})}$$

where $r_{A}$, $r_{B}$, and $r_{O}$ are the ionic radii of A-site cations, B-site cations, and oxygen ions, respectively; t = 1 represents an ideal cubic structure; t < 1 represents orthorhombic or rhombohedral distortions occurring; and >1 represents a hexagonal structure [36].

**Figure 2:**
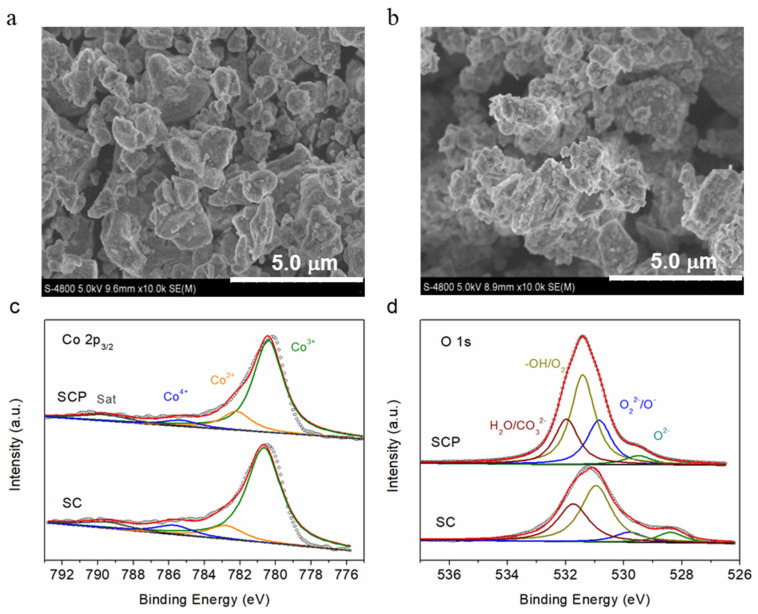
SEM images of SC (**a**) and SCP (**b**) materials. High-resolution Co 2p (**c**) and O 1s (**d**) X-ray photoelectron spectra of SC and SCP materials.

Both SC and SCP materials were tested by a nitrogen adsorption–desorption constant temperature test (BET, Quantachrome, AutoSorb-iO3) and a field emission scanning electron microscope (FE-SEM, Hitachi S-4800) operated at 5 kV. Figure 1b and Figure 1c show that the specific surface areas of SCP and SC are 2.5 and 5.1 cm^2^ g^−1^, respectively. Moreover, the morphology and particle size of the SCP and SC powder, as observed from the SEM images (Figure 2a,b), are not significantly different.

X-ray photoelectron spectroscopy (XPS) was chosen to detect the valence states of transition metals and chemical compositions on the surface of the SCP and SC materials. With the fitted results of the different valences and relative contents in the Co 2p_3/2_ (Figure 2c) and O 1s (Figure 2d) spectra [33,37], it was found that the Co cation existed in the three states of Co^4+^ (785.2 eV), Co^3+^ (780.4 eV), and Co^2+^ (782.5 eV), while O 1s could be fitted to four characteristic peaks, such as lattice oxygen (O^2−^, 529.4 eV), highly oxidative oxygen species (O_2_^2−^/O^−^, 530.8 eV), surface adsorbed oxygen (−OH/O_2_, 531.4 eV), and surface adsorbed water (H_2_O/CO_3_^2−^, 532.0 eV). The fitted results show that the content of high-valence cobalt ions, such as Co^3+^ and Co^4+^, in SCP material is lower than that in SC (listed in Table 1), which can be attributed to the doping of the highly positive charged P^5+^ into SC, leading to part of the high oxidation state of Co^4+^ and/or Co^3+^ being reduced to the trivalent Co^3+^ and even to the divalent Co^2+^ to maintain electroneutrality. Additionally, the O 1s spectra suggests that a much greater amount of the highly oxidative oxygen species (O_2_^2−^/O^−^), which was considered responsible for high catalytic activity, was found for SCP (23.5%) relative to SC (8.5%), which may be the one factor to enhance the good electrocatalytic activities of SCP. The deconvolution results of Co 2p and O 1s XPS peaks analyzed in this work are also consistent with reference [33].

### 2.2. Electrochemical Behaviour of the SC/GCE and SCP/GCE Sensors 

**Figure 3:**
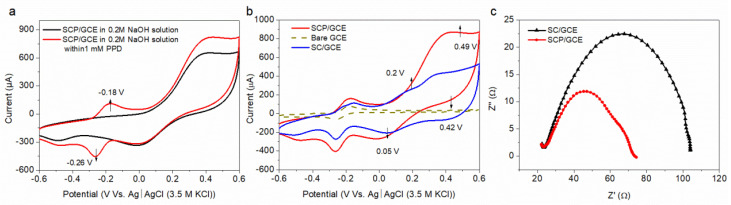
CV profiles for the SCP/GCE sensor tested at a 50 mV s^−1^ scan rate in an Ar-saturated 0.2 M NaOH solution with the presence and/or absence of 1 mM PPD (**a**); CV profiles for SPCE/GCE, SC/GCE and bare GCE sensors in the presence of 1 mM PPD (**b**); EIS for SPCE/GCE and SC/GCE sensors tested in 0.2 M NaOH solution at a frequency of 0.1 Hz to 1 MHz (**c**).

The electrocatalytic activities of SCP and SC for PPD detection were determined by a cyclic voltammetry method (CV) between −0.6 and 0.6 V versus Ag/AgCl (3 M KCl) at a 50 mV s^−1^ scan rate in an Ar-saturated 0.2 M NaOH solution. Figure 3a reveals that a pair of oxidation (at −0.18 V) and reduction (at −0.26 V) reactions occurred for the SCP/GCE sensor in the presence of 1 mM PPD solution, while no obvious electrochemical response was found in an Ar-saturated 0.2 M NaOH solution without PPD. According to our previous work [29], this redox (reduction–oxidation) reaction is the reversible electro-redox process between the PPD and its oxidation product (p-quinonediimine, PQD) on the surface of the electrode. 

In addition, compared with the sensors of SC/GCE and bare GCE, SCP/GCE shows a significant current advantage for the redox reaction of PPD and PQD (Figure 3b). Moreover, the redox peaks of Co^2+^ ↔ Co^3+^ appear at 0.2 V (anodic peak) and 0.05 V (cathodic peak), which are similar for SC/GCE and SCP/GCE, whereas for the redox conversion between Co^3+^ and Co^4+^, appearing at 0.49 V (anodic peak) and 0.42 V (cathodic peak), both the peak currents and peak areas of SCP/GCE are higher than that of SC/GCE, which indicates that the electrical conductivity of SCP is better than SC and that the total amount of Co^3+^ and Co^2+^ with low oxidation states in SCP is more than that in SC (consistent with the XPS results in Table 1). 

Moreover, the surface and interfacial characterization of SCP/GCE and SC/GCE can also be confirmed in our work by electrochemical impedance spectroscopy (EIS), which was tested in 0.2 M NaOH solution over a frequency range of 0.1 Hz to 1 MHz. The semicircle portion at high frequency represents the charge transfer limited process [38,39], as shown in Figure 3c, and the semicircle value of SPC/GCE is smaller than that of SC/GCE, suggesting the faster electron transfer rate of SPC/GCE. 

**Figure 4:**
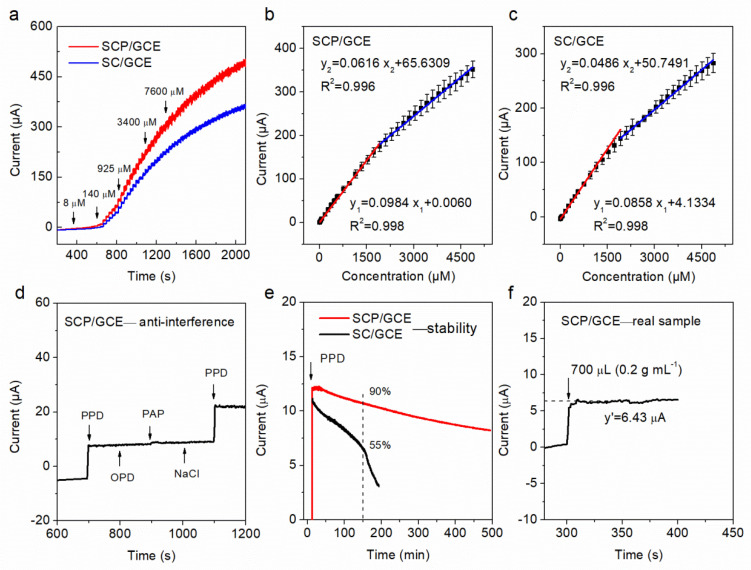
Chronoamperometric responses (I–t) for SC/GCE and SCP/GCE obtained in an Ar-saturated 0.2 M NaOH solution with a continuous injection of PPD (**a**); corresponding current–concentration (I–C) linear fitting results for (**b**) SCP/GCE and (**c**) SC/GCE; anti-interference (**d**), stability (**e**), and real sample tests (**f**) of the SCP/GCE electrode.

The chronoamperometric method (I–t) was used to test the electrocatalytic activities and sensing performance of SCP/GCE and SC/GCE sensors towards PPD detection at a constant potential of −0.18 V, which is the oxidation potential of PPD to PQD according to the CV results (Figure 4a). Different concentrations of PPD were added into an Ar-saturated 0.2 M NaOH electrolyte every 30 s in a continuous stirring system (300 rpm). The test results reveal that the liner determination ranges, sensitivity, response time, and limit of determination (LOD) of SCP/GCE are 0.8–5000 μM, 502 (0.8–2000 μM) and 314 (2000–5000 μM) μA mM^−1^ cm^−2^, 3 s, and 0.3 μM, respectively. In addition, the linear regression equations of the SCP/GCE sensor are I_1_ (mA) = 0.0984·C_1_ (μM) + 0.0060 (R^2^ = 0.998) and I_2_ (mA) = 0.0616·C_2_ (μM) + 65.6309 (R^2^ = 0.996), corresponding to the range of 0.8–2000 μM (C_1_) and 2000–5000 μM (C_2_), respectively. As shown in Table 2 and Figure 4b,c, all of these sensing parameters of the SCP/GCE sensor are better than those of the SC/GCE, demonstrating that the electrocatalytic activity of SCP for PPD detection is higher than that of SC. 

**Figure 5:**
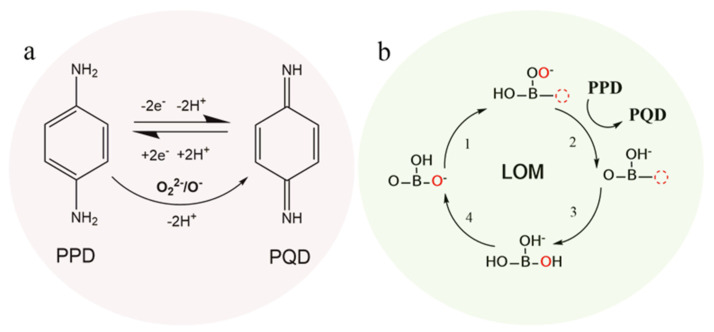
Oxidation effect of O_2_^2−^/O^−^ for PPD to PQD and the reversible electro-redox reaction mechanism between PPD and PQD (**a**); oxidation activity for PPD by the LOM (lattice oxygen mechanism with high active species of lattice O vacancies and adsorbed –OO) theory (**b**).

Therefore, 5 mol% P doping in SC (SCP) definitely enhances the catalytic activity and electrochemical detection performance for PPD compared to the non-doped one (SC). The first contributing factor is the better electron conductivity of SCP. With P doping, some of the Co^4+^ was reduced to higher spin Co^3+^ due to its narrower gap between *e_g_* and *t*_2*g*_ in the Co 3d state, thereby improving the electrical conductivity of the coating [40]. Additionally, from the aspect of structure, the bond angle of B-O-B (B is Co or P) for the tetragonal structure of SCP is close to 180°, which has a smaller local strain than SC and, thus, facilitates good electron conductivity [41]. The second contributing factor is the large amount of highly oxidative oxygen species O_2_^2−^/O^−^, which not only have a high electrocatalytic activity for water splitting and fuel cells [42,43], but are also good for electrochemical sensors such as those detecting glucose and hydrogen peroxide [44]. Here, the PPD is first oxidized to PQD by the active species of O_2_^2−^/O^−^, which then, in turn, supports the reversible two-electron redox reaction between PPD and PQD and finally improves the current response signal (the process is shown in Figure 5a). 

The third contributing factor may be the lattice oxygen mechanism (LOM) with highly active species of lattice O vacancies and adsorbed –OO, which results from strong metal–oxygen covalent bonds, such as the high valence state of Co^3+^/Co^4+^ with oxygen bonds [45,46,47]. Therefore, the SCP with P^5+^ doping could produce more active sites on the P^5+^-oxygen bonds. Moreover, considering the reaction energy and overpotential for oxygen evolution reaction, density functional theory (DFT) calculations have proved that the LOM exhibits highly active effects for electrocatalytic oxidation reactions without valence changes to B-site cations [48,49]. In addition, the high electrocatalytic effect of the LOM mechanism for glucose and H_2_O_2_ oxidation and detection has been proposed in our previous work [44]. Herein, we propose that the LOM electrocatalytic mechanism is also suitable for the PPD oxidation reaction, as shown in Figure 5b. 

### 2.3. Anti-Interference, Stability, Repeatability, and Real Sample Detection of SCP/GCE

The anti-interference, stability, and repeatability of SCP/GCE were evaluated in our work, respectively. For the anti-interference test, the possible signal influence from interfering species, including o-phenylenediamine (100 μM), p-aminophenol (100 μM), and sodium chloride (100 μM), was tested by recording the response current. Notably, the above tests displayed no significant interference in the determination of PPD (100 μM) (Figure 4d). In addition, Figure 4e shows that the SCP/GCE can keep 90% of the initial current signal, while SC/GCE only has 55% of the initial current signal at the same conditioner (−0.18 V, 300 rpm, and 0.2 M NaOH within 100 μM PPD) after 150 min of continuous testing the system, which indicates good stability of SCP/GCE. It can be explained that the P doping effectively reduces the leaching of Sr and, thus, greatly decreases the thickness of the amorphous layer that forms on the surface of the catalyst relative to that of SC. The amorphous layer covers the active sites and blocks the diffusion of molecules, and also has a low electrical conductivity, thereby significantly impacting the catalytic activity and stability of the sensors.

Additionally, it is well known that the repeatability of sensors is vitally important for practical applications. Here, five different SCP/GCE electrodes in the same content solution (1 mM PPD, an Ar-saturated 0.2 M NaOH) were tested individually (Table 3). The standard error of repeatability does not exceed 2.3%, suggesting that SCP/GCE has outstanding repeatability and a great research potential for PPD determination. 

For real sample detection to determine the concentration of PPD, a filtrate solution of hair dye (Bigen, natural black) was chosen. Approximately 700 μL of initial solution (0.2 g L^−1^) was added into 100 mL of an Ar-saturated 0.2 M NaOH solution with continuous stirring. The current response rapidly increases to approximately 6.43 μA (as shown in Figure 4f); thus, the concentration of PPD in the mixing solution is about 9390 μM, which is calculated by the linear regression equation of I_1_. Besides, the standard content of the real sample is about 8877 μM, as verified by the results of ultraviolet–visible spectrophotometry (UV–vis) [29]. Therefore, the error of the proposed SCP/GCE sensor is no more than 5.78%, indicating that a high reliability of SPC/GCE is achieved which is beneficial for practical applications. 

## 3. Experiments

### 3.1. Reagents and Instruments

Co(NO_3_)_2_·6H_2_O, Sr(NO_3_)_2_, NaOH, (NH_4_)H_2_PO_4_, NH_3_·H_2_O, citric acid (CA), and C_2_H_5_OH were purchased from Sinopharm Group Chemical Reagent. Ethylenediaminetetraacetic acid (EDTA) and p-phenylenediamine (PPD) were purchased from Shanghai Lingfeng and Aladdin Chemical Reagent, respectively. Bigen (Natural Black, 881) was chosen as the real sample and was made in Nagoya, Aichi, Japan. Deionized (DI) water was used to prepare all solutions.

All electrochemical experiments were undertaken on a CHI760E electrochemical workstation (Shanghai Chenhua Co., Ltd., Shanghai, China). Furthermore, a three-electrode system was employed to perform the electrochemical measurement. The modified glass carbon electrode, carbon rod, and Ag/AgCl (3 M KCl) were used as the working electrode, counter electrode, and reference electrode, respectively.

### 3.2. Synthesis of SrCoO_3−δ_ and SrCo_0.95_P_0.05_O_3−δ_ Perovskite Oxides

In this experiment, the perovskite oxides of SrCoO_3−δ_ and SrCo_0.95_P_0.05_O_3−δ_ were synthesized by a sol–gel process using CA and EDTA as complexing agents. First, all metal nitrates and (NH_4_)H_2_PO_4_ were dissolved in DI water in an exact stoichiometric ratio. A certain amount of EDTA and CA was then added into the above mixed solution (the molar ratio of CA: EDTA: total metal ions is 2:1:1). The pH value of the solution was adjusted to 6–7 by NH_3_·H_2_O. A purple and transparent gel solution was obtained after continuously stirring at 90 °C for 2–3 h, which was then heated at 200 °C in an air oven for 5–6 h to dry the gel and obtain a fluffy precursor. Finally, the SrCoO_3−δ_ and SrCo_0.95_P_0.05_O_3−δ_ perovskite crystallized structures were successfully formed after heating at 1100 ℃ for at least 6 h in a muffle furnace.

### 3.3. Preparation of the Working Electrodes 

First, the glass carbon electrode (GCE, 0.196 cm^2^) was polished with 0.3 and 0.05 μm of α-Al_2_O_3_ powder for 10 min, successively. Then, the GCE was completely cleaned with DI water and ethanol several times. Secondly, 20 mg of perovskite catalysts, 10 mg of conductive carbon powder (Super P Li), and 0.1 mL of 5 wt.% Nafion^@^ solution were mixed into 0.9 mL of DI water and subjected to sonication for 1 h to obtain homogeneous catalyst inks. Finally, 5 µL of the catalyst ink was dispersed on the GCE surface (0.196 cm^2^) and dried at room temperature for 30 min to obtain the SC/GCE and SCP/GCE sensors.

### 3.4. Electrochemical Measurements

Cyclic voltammetry (CV) and chronoamperometric (I–t) methods were used to analyze and detect PPD due to their high sensitivity and fast response characteristics. The chronoamperometric method was used to test the sensitivity, anti-interference, stability, repeatability, and real sample detection performance of PPD in an Ar-saturated 0.2 M NaOH solution with a 300 rpm rotation rate and a constant potential support. 

## 4. Conclusions

In this study, a small content of non-metallic element P was doped into SrCoO_3_ (SC) to form an excellent perovskite material, SrCo_0.95_P_0.05_O_3−δ_ (SCP), which is applied for the first time as a catalyst for p-phenylenediamine (PPD) detection. SCP has higher electrocatalytic activity compared to the parent SC. The stabilized tetragonal structure, high electrical conductivity, and large amount of highly oxidative oxygen species (O_2_^2−^/O^−^) are the contributing factors to the good PPD detection of SCP. In particular, the stability of the SCP/GCE sensor is greatly enhanced due to the P doping, which can reduce the formation of an amorphous layer on the surface of SCP that is caused by the leaching of Sr. Furthermore, the lattice oxygen mechanism (LOM) with highly active species of lattice O vacancies and adsorbed –OO is beneficial to the oxidation of PPD to PQD. Additionally, good anti-interference and repeatability, as well as a high reliability of the SCP/GCE sensor, suggest that it has great potential for the detection of real samples. Thus, the following step is to further improve and modify the SCP/GCE sensor to reduce the cost and increase the accuracy for practical product detection, such as hair dyes or environmental monitoring.

## Figures and Tables

**Table 1:** Relative amounts of Co^2+^, Co^3+^, and Co^4+^ on the SC and SCP surfaces calculated by deconvolution of the Co 2p and O 1s peaks shown in Figure 2c,d.

Sample	Co 2p_3/2_			O 1s			
	Co^4+^ (%)	Co^3+^ (%)	Co^2+^ (%)	Lattice Oxygen O^2−^ (%)	Highly Oxidative Oxygen Species O_2_^2−^/O^−^ (%)	Surface Adsorbed Oxygen −OH/O_2_ (%)	Surface Adsorbed Water H_2_O/CO_3_^2−^ (%)
SCP	5.9	78.3	15.8	5	23.5	47.4	24.1
SC	8.4	80	11.6	8.1	8.5	49.7	33.7

**Table 2:** Sensing performances of SC/GCE, SCP/GCE, and other sensors toward PPD detection.

Samples	Detection Method	LOD (µM)	Liner Response Range (µM)	Sensitivity (µA mM^−1^ cm^−2^)	Response Time (s)	Ref
SCP/GCE	Electrochemistry	0.3	0.8–5000	502 (0.8–2000 µM) 314 (2000–5000 µM)	3	This work
SC/GCE	Electrochemistry	0.5	1.5–5000	438 (1.5–2000 µM) 248 (2000–5000 µM)	4	This work
HRP/NPG/GCE	Electrochemistry	0.33	2–170	26.5		[18]
PSC82/GCE	Electrochemistry	0.17	0.5–2900	655	3	[29]
P-TiO_2_NTs	Electrochemistry	0.056	0.5–98.6	1456 (0.5–5µM)		[12]
Fe–SAs@FNC	Colorimetry	0.07	0.2–50			[50]
Fe_3_O_4_/N-GQDs	Colorimetry	0.53	2–70			[51]
ZnBNC SAzyme	Colorimetry	0.1	0.3–10			[52]
AgNPs	Colorimetry	0.53	0.002–1500			[53]
AgNPs layer	Surface-enhanced Raman spectroscopy	10^−11^	10^−10–^10^−6^			[4]
S-PPD-DCM	Fluorescence	0.05	0.09–0.92			[9]
BCP-Py-CHO	Fluorescence	0.007	0.02–1.5			[54]
CDs@NBD	Fluorescence	0.056	0.1–10			[55]

**Table 3:** Variation in the measured concentrations of an Ar-saturated 0.2 M NaOH solution containing 1 mM of PPD from five measurements using five different SCP/GCE sensors.

Electrode Number	1	2	3	4	5	Average Value	Amount Added (Mm)	Standard Error (%)	Deviation (%)
Result/(mM)	0.978	0.975	0.985	0.997	1.031	0.993	1	2.3%	0.7%

## Data Availability

Not applicable.
